# Hypertension and the risk of endometrial cancer: a systematic review and meta-analysis of case-control and cohort studies

**DOI:** 10.1038/srep44808

**Published:** 2017-04-07

**Authors:** Dagfinn Aune, Abhijit Sen, Lars J. Vatten

**Affiliations:** 1 Department of Epidemiology and Biostatistics, Imperial College, London, UK; 2 Department of Public Health and General Practice, Faculty of Medicine, Norwegian University of Science and Technology, Trondheim, Norway.; 3 Bjørknes University College, Oslo, Norway.

## Abstract

A history of hypertension has been associated with increased risk of endometrial cancer in several studies, but the results have not been consistent. We conducted a systematic review and meta-analysis of case-control and cohort studies to clarify the association between hypertension and endometrial cancer risk. PubMed and Embase databases were searched up to 27^th^ of February 2016. Prospective and case-control studies which reported adjusted relative risk estimates and 95% confidence intervals of endometrial cancer associated with a hypertension diagnosis were included. Summary relative risks were estimated using a random effects model. Nineteen case-control studies and 6 cohort studies were included. The summary RR was 1.61 (95% CI: 1.41–1.85, I^2^ = 86%) for all studies, 1.73 (95% CI: 1.45–2.06, I^2^ = 89%) for case-control studies and 1.32 (95% CI: 1.12–1.56, I^2^ = 47%) for cohort studies. The association between hypertension and endometrial cancer was weaker, but still significant, among studies with adjustment for smoking, BMI, oral contraceptive use, and parity, compared to studies without such adjustment. This meta-analysis suggest an increased risk of endometrial cancer among patients with hypertension, however, further studies with more comprehensive adjustments for confounders are warranted to clarify the association.

Hypertension is a major cause of morbidity and mortality worldwide and is an established risk factor for coronary heart disease and stroke^1^,^2^. Globally a high systolic blood pressure accounted for 10.4 million deaths and 208.1 million disability-adjusted life-years (DALYs) in 2013^3^. Important risk factors for hypertension include overweight and obesity^4^, low physical activity^5^,^6^, high alcohol consumption^7^, dietary factors^8^,^9^,^10^,^11^, and use of non-narcotic analgesics^12^.

Endometrial cancer is the eighth most common type of cancer in women with approximately 320 000 cases recorded in 2012, accounting for about 4.8% of all cancers in women (2.3% overall)^13^. It is more common in high-income countries than in low-income countries, however, its incidence has been increasing in populations undergoing urbanization and economic growth, in parallel with increasing obesity rates and sedentary lifestyles^14^,^15^. Several risk factors for endometrial cancer have been established including excess body weight^16^, low physical activity^17^, diabetes history^18^, and use of unopposed hormone replacement therapy^19^. A history of hypertension has been evaluated as a risk factor for endometrial cancer in several case-control^20^,^21^,^22^,^23^,^24^,^25^,^26^,^27^,^28^,^29^,^30^,^31^,^32^,^33^,^34^,^35^,^36^,^37^,^38^ and cohort studies^39^,^40^,^41^,^42^,^43^,^44^, and many^20^,^21^,^24^,^25^,^26^,^28^,^30^,^32^,^33^,^34^,^35^,^36^,^37^,^38^,^39^,^42^,^44^, but not all^22^,^23^,^27^,^29^,^31^,^39^,^42^,^44^ of these found an increased endometrial cancer risk. Because obesity and diabetes are important risk factors for both hypertension^45^,^46^ and endometrial cancer^16^,^18^ it is not clear whether the association between hypertension and endometrial cancer could be due to confounding by these factors because some studies did not adjust for BMI^20^,^21^,^25^,^33^,^35^,^38^ or diabetes^20^,^21^,^24^,^25^,^28^,^29^,^33^,^35^,^38^. We conducted a systematic review and meta-analysis of case-control and cohort studies that had investigated the association between hypertension and endometrial cancer risk with an aim of clarifying the strength of the association, possible sources of heterogeneity and potential confounding by other risk factors.

## Methods

### Search strategy and inclusion criteria

We searched the PubMed and Embase databases up to 27^th^ February 2016 for eligible studies. We used the following search terms in the PubMed search: (hypertension OR high blood pressure OR blood pressure OR risk factor) AND (endometrial cancer OR uterine cancer). We followed standard criteria for reporting meta-analyses^47^.

#### Study selection

We included published retrospective case-control studies and cohort studies that investigated the association between hypertension and the risk of endometrial cancer. Adjusted estimates of the relative risk (odds ratios and hazard ratios which were considered to be approximately equal given that endometrial cancer is a relatively uncommon cancer) had to be available with the 95% CIs in the publication. A list of excluded studies and exclusion reasons is provided in Supplementary Table 1. DA and AS conducted the study selection.

#### Data extraction

The following data were extracted from each study: The first author’s last name, publication year, country where the study was conducted, study period, sample size, number of cases/controls, exposure and subgroups of tumor characteristics (low, moderate or high aggressiveness) or cancer type (type 1 vs. type 2), relative risks and 95% confidence intervals for the association and variables adjusted for in the analysis. Data were extracted by one reviewer (DA) and checked for accuracy by a second reviewer (AS).

### Statistical methods

We calculated summary relative risks of developing endometrial cancer by history of hypertension using the random-effects model by DerSimonian and Laird^48^ which takes into account both within and between study variation (heterogeneity). The average of the natural logarithm of the relative risks was estimated and the relative risk from each study was weighted by the inverse of its variance^49^.

Heterogeneity between studies was evaluated using Q and I^2^ statistics^50^. Cochran’s Q is calculated as the weighted sum of squared differences between individual study effects and the pooled effects across studies, with weights being those in the pooling method. I^2^ is a measure of how much of the heterogeneity that is due to between study variation rather than chance. I^2^-values of 25%, 50% and 75% indicates low, moderate and high heterogeneity respectively. We conducted main analyses (all studies combined) and stratified by study design (cohort studies, case-control studies) because of the greater potential for recall and selection bias in retrospective case-control studies and to investigate sources of potential heterogeneity. We also conducted subgroup analyses by other study characteristics such as sample size, number of cases, geographic location, and by adjustment for confounding factors. We also conducted a stratified analysis by whether the articles explicitly stated that participants with prevalent hysterectomies at baseline were excluded, and/or whether participants with incident hysterectomies were censored during follow-up in cohort studies, or excluded from the control group in case-control studies.

Publication bias was assessed using Egger’s test^51^ and Begg-Mazumdar’s test^52^ and by inspection of funnel plots. Study quality was assessed using the Newcastle-Ottawa scale which ranks the studies on a scale from 0 to 9 based on the selection of the study population, comparability between cases and non-cases and the assessment of the outcome^53^. The statistical analyses were conducted using the software package Stata, version 13.0 software (StataCorp, Texas, US).

## Results

**Figure 1:**
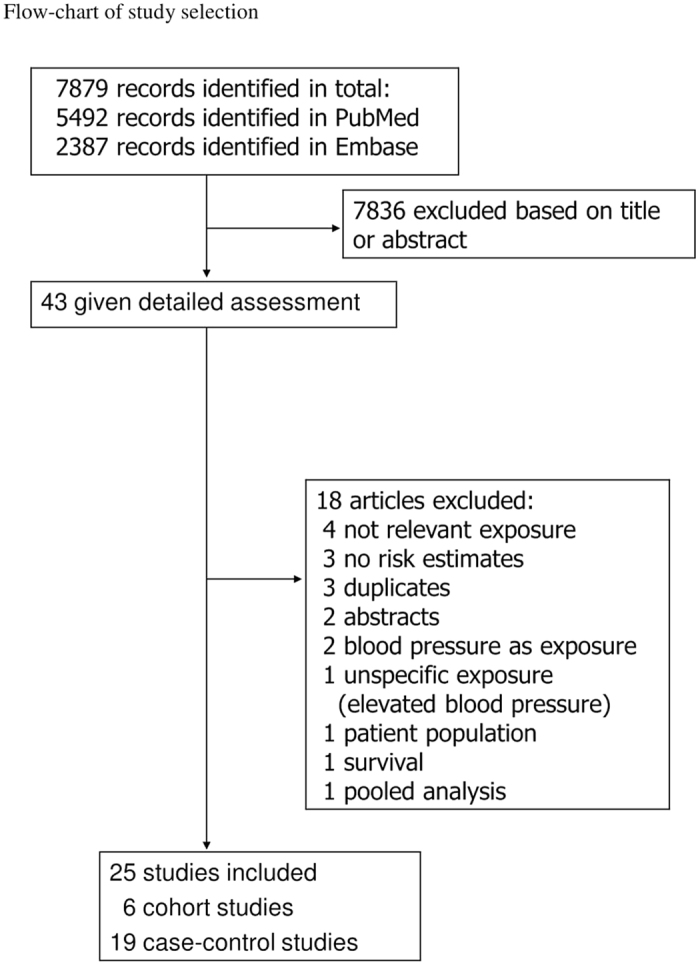
Flow-chart of study selection.

Out of a total 7879 records identified by the search we included 25 studies with 28385 cases and 300598 participants in the meta-analysis of hypertension and endometrial cancer risk, including six cohort studies^39^,^40^,^41^,^42^,^43^,^44^ and nineteen case-control studies^20^,^21^,^22^,^23^,^24^,^25^,^26^,^27^,^28^,^29^,^30^,^31^,^32^,^33^,^34^,^35^,^36^,^37^ (Fig. 1 and Tables 1 and 2). Fourteen of the studies were from North-America, seven were from Europe, and four were from Asia (Tables 1 and 2).

**Figure 2:**
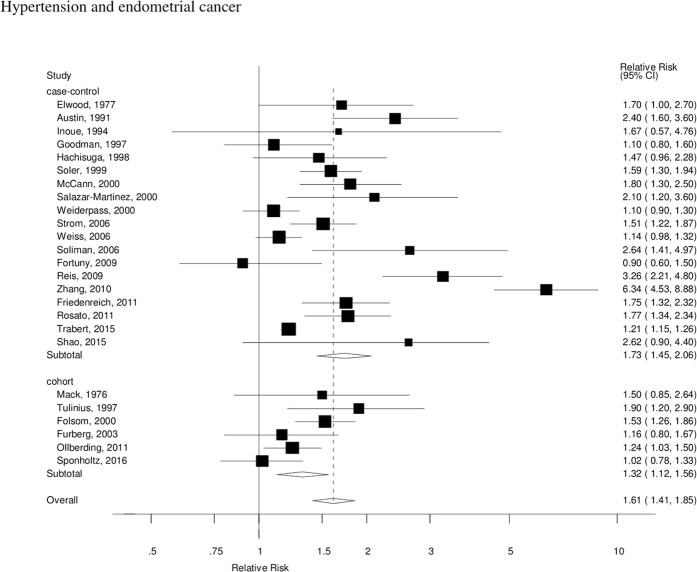
Hypertension and endometrial cancer, forest plot.

**Figure 3:**
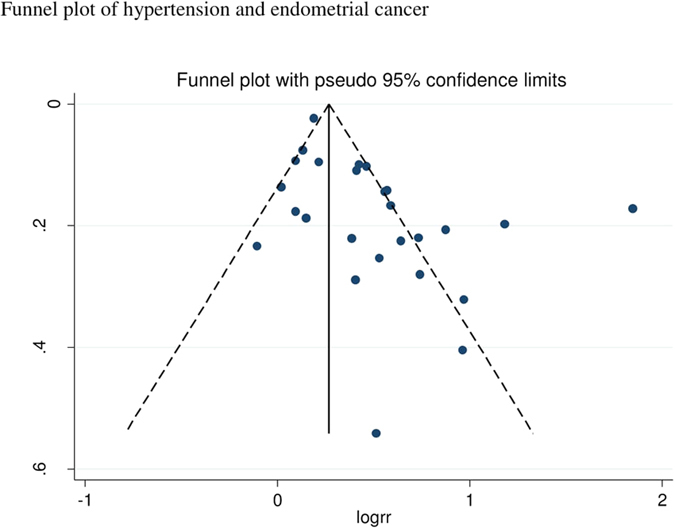
Hypertension and endometrial cancer, funnel plot.

The summary RR for all studies was 1.61 (95% CI: 1.41–1.85, I^2^ = 86%), and it was 1.73 (95% CI: 1.45–2.06, I^2^ = 89%) for case-control studies and 1.32 (95% CI: 1.12–1.56, I^2^ = 47%) for cohort studies (Fig. 2), however, the test for heterogeneity by study design was not significant, p = 0.19. In sensitivity analyses excluding one study at a time the summary RR ranged from 1.49 (95% CI: 1.34–1.65) when excluding the study by Zhang *et al*.^33^ to 1.65 (95% CI: 1.41–1.94) when excluding the study by Trabert *et al*.^36^. There was evidence of publication bias with Egger’s test, p = 0.005 (Fig. 3), however, when stratified by study design this was observed among case-control studies, p = 0.007, but not among cohort studies, p = 0.78.

### Subgroup and sensitivity analyses, study quality assessment

There were positive associations in almost all subgroup analyses (Table 3), and although there was no heterogeneity when stratified by study design, geographic location or number of cases, there was indication of heterogeneity when studies were stratified by confounding factors including smoking (p = 0.02), BMI (p = 0.003), oral contraceptive use (p = 0.02), hormone replacement therapy (p = 0.08), parity (p = 0.03), and age at menopause (p = 0.07), with weaker, but still significant associations among studies with such adjustments. When we conducted sensitivity analyses removing one study at a time, the size of the summary estimate persisted and did not vary substantially (Supplementary Table 2).

In a further sensitivity analysis we also conducted a subgroup analysis by whether the studies explicitly stated that they excluded participants with prevalent hysterectomies at baseline and/or stated that they censored participants at the time of incident hysterectomy (cohort studies) or excluded participants who had undergone hysterectomy from the control group (case-control studies). The summary RR was 1.51 (95% CI: 1.28–1.78, I^2^ = 88.5%) for studies with such exclusions or censoring and 1.81 (95% CI: 1.49–2.20, I^2^ = 56.5%) for studies without such exclusions or censoring.

In a sensitivity analysis we also included a pooled analysis which assessed the association between quintiles of systolic blood pressure and endometrial cancer risk^54^, using the relative risk for the highest vs. the lowest quintile of systolic blood pressure. The results were not materially altered, summary RR = 1.61 (95% CI: 1.42–1.83, I^2^ = 38%) for all studies and 1.33 (95% CI: 1.16–1.52, I^2^ = 86%) for cohort studies. Further including another cohort study^55^ which reported on elevated blood pressure (≥130/≥85 vs. <130/<85 mm/Hg) or self-reported hypertension, not only hypertension, did also not substantially alter the results, summary RR = 1.57 (95% CI: 1.38–1.78, I^2^ = 85%) for all studies and summary RR = 1.28 (95% CI: 1.12–1.48, I^2^ = 46%) for cohort studies. Mean (median) study quality scores were 7.3 (7.0) for all studies combined, 7.3 (7.0) for case-control studies, and 7.3 (7.0) for cohort studies).

## Discussion

To our knowledge this is the first meta-analysis of published observational studies of hypertension and the risk of endometrial cancer and our results confirm that hypertension is a strong risk factor for endometrial cancer with a 61% increase in the relative risk, however, the association was weaker in cohort studies (RR = 1.32) than among case-control studies (RR = 1.73). These findings are consistent with a large cohort study of 290 000 women in Austria, Norway and Sweden which found an increased endometrial cancer risk with increasing levels of diastolic blood pressure and in particular, systolic blood pressure^54^. The results also persisted in a sensitivity analysis including the results from this cohort study^54^ as well as the EPIC study^55^, which reported on elevated blood pressure or hypertension.

The present meta-analysis has some limitations. As hypertension is a condition that is strongly related to lifestyle factors and some medical conditions including diet, BMI, physical activity, and diabetes we cannot entirely exclude the possibility that the observed association between hypertension and endometrial cancer risk at least partly could be due to confounding. We found that the association was weaker, but still statistically significant, among studies that adjusted for smoking, BMI, oral contraceptive use, hormone replacement use, parity and age at menopause (RR = 1.14–1.34 for studies with such adjustment vs. 1.74–2.10 for studies without such adjustment). However, because there was still a significant association in subgroups that adjusted for these factors it could indicate that there is an adverse effect of hypertension on endometrial cancer risk, but that it may be slightly weaker than what was suggested from the overall summary estimates. Because the original studies did not stratify for BMI or diabetes it was not possible for us to investigate whether the association was limited to specific weight classes or if it was modified by diabetes status.

We also found that the positive association between hypertension and endometrial cancer persisted when the studies were stratified by whether participants with prevalent hysterectomies at baseline were excluded and/or whether participants with incident hysterectomies were censored, or whether prevalent hysterectomies were excluded from the control group. Hypertension may also be related to hysterectomies^56^,^57^,^58^, and could potentially bias the risk estimates, however, any bias would most likely be toward the null. We cannot exclude the possibility of residual confounding from other risk factors such as use of intrauterine device^59^, polycystic ovarial syndrome^60^, or other potential risk factors that the original studies may not have adjusted for.

Case-control studies are more likely to be affected by certain biases, such as recall bias and selection bias. Because we included both case-control and cohort studies there is a possibility that recall or selection bias might have affected the results in the case-control studies and the overall summary estimate. Although the association appeared to be stronger in case-control studies than among cohort studies, there was still a significant association among cohort studies, which suggest that recall bias or selection biases does not entirely explain the observed association. In addition, there was some indication of publication bias with Egger’s test, but this appeared to be restricted to the analyses of case-control studies and all studies combined, and was not observed among the cohort studies.

The biological mechanism(s) that may explain an adverse effect of hypertension on endometrial cancer risk are unclear at present. It has been suggested that long-term hypertension may lead to cellular senescence and inhibition of apoptosis^61^. It has also been suggested that medications used for the treatment of hypertension could increase cancer risk, however, a meta-analysis found little evidence of an association with overall cancer^62^, and a cohort study found no relation with female genital cancers^63^, although few studies have specifically investigated endometrial cancer.

Strengths of the present meta-analysis include the comprehensive search strategy, the detailed subgroup and sensitivity analyses, and the large sample size providing a more robust estimate of the association between hypertension and endometrial cancer risk. To date relatively few studies have investigated the association between hypertensive disorders of pregnancy and endometrial cancer risk with one study suggesting an increased risk with hypertensive disorders overall^64^, while another study found no association with preeclampsia overall, although an increased risk was observed with early-onset preeclampsia^65^. Any further studies could better assess the causality of the observed association between hypertension and endometrial cancer by using genetic risk scores for hypertension^66^,^67^. In addition, clarification of potential effect modification by age at exposure, BMI and diabetes status, and further studies of the association with subtypes of endometrial cancer are needed.

In conclusion, the results from this systematic review and meta-analysis suggest that women with hypertension may have a 61% increase in the relative risk of developing endometrial cancer. Any further studies should clarify potential effect modification by age, BMI and diabetes status, and the causality of the observed association, as well as the potential underlying mechanism(s).

## Additional Information

**How to cite this article:** Aune, D. *et al*. Hypertension and the risk of endometrial cancer: a systematic review and meta-analysis of case-control and cohort studies. *Sci. Rep.* **7**, 44808; doi: 10.1038/srep44808 (2017).

**Publisher's note:** Springer Nature remains neutral with regard to jurisdictional claims in published maps and institutional affiliations.

## Supplementary Material

Supplementary Information

## Figures and Tables

**Table 1:** Prospective studies of hypertension and endometrial cancer.

First author, publication year, country	Number of participants, age, number of cases	Study period	Assessment of hypertension	Cut-off for hypertension	Exposure	Comparison	Relative risk (95% confidence interval)	Adjustment for confounders
Mack T *et al*., 1976, USA	Nested case-control study: 63 cases 252 controls	1971–1975, ~4 years follow-up	Self-reported	Not available	Hypertension	Yes vs. no	1.50 (0.85–2.64)	Age, marital status, community
Tulinius H *et al*., 1997, Iceland	11580 women, mean age 50.5 years: 98 cases	1968–1995, ~15.1 years follow-up	Measured	Not available	Hypertension	Yes vs. no	1.9 (1.2–2.9)	Age
Folsom AR *et al*., 2003, USA	23335 women, age 55–69 years: 415 cases	1986–2000, 15.7 years follow-up	Self-reported	Not available	Hypertension	Yes vs. no	1.53 (1.26–1.86)	Age
Furberg AS *et al*., 2003, Norway	24460 women, age 20–49 years: 130 cases	1974–1981–1996, 15.7 years follow-up	Measured (mercury sphygmo-manometer)	≥140/≥90 mmHg	Hypertension	Consistently normotensive	1.00	Age, geographical region, height, BMI, recreational and occupational activity, smoking, parity
						Hypertensive in one survey	1.11 (0.70–1.77)	
						Consistently hypertensive	1.24 (0.69–2.25)	
Ollberding NJ *et al*., 2011, USA	46027 postm. Women, age 45–75 years: 489 cases	1993/1996–2007, 13.6 years follow-up	Self-reported	Not available	Hypertension	Yes vs. no	1.24 (1.03–1.50)	Age, race/ethnicity, age at cohort entry, total calories, BMI, age at menarche, age at menopause, parity, duration of OC use, HRT use, smoking status, diabetes
Sponholtz TR *et al*., 2016, USA	47577 women, age 21–69 years: 274 cases	1995–2013, 14 years follow-up	Self-reported	Not available	Hypertension	Yes vs. no	1.02 (0.78–1.33)	Age, study period, age at menarche, parity, menopausal status, OC use, estrogen-only hormone use, estrogen plus progestin hormone use, smoking status, BMI, vigorous physical activity, statin use, metformin use

BMI = body mass index, OC use = oral contraceptive use, HRT use = hormone replacement therapy use.

**Table 2:** Case-control studies of hypertension and endometrial cancer.

First author, publication year, country	Number of cases and controls, age	Study period	Assessment of hypertension	Cut-off for hypertension	Exposure, subgroup, outcome	Comparison	Relative risk (95% confidence interval)	Adjustment for confounders or matching variables
Elwood JM *et al*., 1977, USA	212 cases 1198 population controls Age 55–69 years	1965–1969	Self-reported	Not available	Hypertension	Yes vs. no	1.7 (1.0–2.7)	Year of birth
Austin H *et al*., 1991, USA	168 cases 334 hospital controls Age 40–82 years	1985–1988	Self-reported (interview)	Not available	Hypertension	Yes vs. no	2.4 (1.6–3.6)	Age, race, years of schooling
Inoue M *et al*., 1994, Japan	143 cases 143 hospital controls Age 22–79 years	1979–1992	Medical records	Not available	Hypertension	Yes vs. no	1.67 (0.57–4.76)	Age, obesity, personal cancer history, diabetes mellitus, parity
Goodman MT *et al*., 1997, USA	332 cases 511 population controls Age 18–84 years	1985–1993	Self-reported (interview	Not available	Hypertension	Yes vs. no	1.1 (0.8–1.6)	Age, ethnicity, pregnancy history, OC use, unopposed estrogen use, diabetes history, BMI
Hachisuga T *et al*., 1998, Japan	242 cases 1021 hospital controls Age 20–79 years	1980–1989	Medical records	Not available	Hypertension	Yes vs. no	1.47 (0.96–2.28)	Age, parity, BMI, diabetes mellitus
Soler M *et al*., 1999, Italy	745 cases 3054 hospital controls Age <75 years	1983–1996	Self-reported (interview)	Not available	Treated hypertension	Yes vs. no	1.59 (1.30–1.94)	Age, area of residence, education, smoking, alcohol, parity, menopausal status, BMI
McCann SE *et al*., 2000, USA	232 cases 639 population controls Age 40–85 years	1986–1991	Self-reported (interview)	Not available	Hypertension	Yes vs. no	1.8 (1.3–2.5)	Age
Salazar-Martinez E *et al*., 2000, Mexico	85 cases 668 population controls Mean age 61.7/60.2 years	1995–1997	Self-reported (interview)	Not available	Hypertension	Yes vs. no	2.1 (1.2–3.6)	Age, anovulatory index, smoking, physical activity, menopausal status, diabetes, BMI
Weiderpass E *et al*., 2000, Sweden	709 cases 3368 population controls Age 50–74 years	1994–1995	Self-reported	Not available	Hypertension	Yes vs. no	1.1 (0.9–1.3)	Age, age at menarche, parity, age at last birth, age at menopause, smoking, OC use, HRT, diabetes mellitus, recent BMI
Strom BL *et al*., 2006, USA	511 cases 1412 population controls Age 50–79 years	1999–2002	Self-reported (interview)	Not available	Hypertension	Yes vs. no	1.51 (1.22–1.87)	Age, ethnicity, education, BMI, number of full-term pregnancies, years of menses, type of menopause, smoking status, years of smoking, OC use
Weiss JM *et al*., 2006, USA	1304 cases 1779 population controls Age 45–74 years	1985–1991	Self-reported (interview)	Not available	Hypertension, low tumor aggressiveness	Yes vs. no	1.2 (1.0–1.6)	Age, HRT, BMI, county of residence, referent year
		1994–1995			Hypertension, moderate tumor aggressiveness	Yes vs. no	1.1 (0.9–1.4)	
		1997–1999			Hypertension, high tumor aggressiveness	Yes vs. no	1.1 (0.7–1.6)	
Soliman PT *et al*., 2006, USA	117 cases 238 hospital controls Age 25–88 years	2000–2004	Medical records	Not available	Hypertension	Yes vs. No	2.64 (1.41–4.97)	Age, BMI, diabetes
Fortuny J *et al*., 2009, USA	469 cases 467 population controls Age ≥21 years	2001–2005	Self-reported (interview)	Not available	Hypertension	Yes vs. no	0.9 (0.6–1.5)	Age, BMI, education, race, age at menarche, HRT, OC use, age at menopause, parity, smoking, FH – EC, type 2 diabetes, biguanides, insulin, sulphonylureas, hypercholesterolemia, statins, fibrates, ACE-inhibitors, beta-blockers, calcium channel blockers, angiotensin 2 receptor antagonists, thiazide diuretics, loop diuretics, K sparing diuretics, osteoporosis, biphosphonates, calcitonin, endometrial cancer fibroids
Reis N *et al*., 2009, Turkey	285 cases 1050 hospital controls Age 43–76 years	2002–2003	Self-report of treated hypertension or physician-diagnosis (interview)	Not available	Hypertension	Yes vs. no	3.26 (2.21–4.80)	Age, education, diabetes, parity, age at menarche, HRT use, 1st degree relative history of breast, endometrial cancer or colorectal cancer, 2nd degree relative with history of breast and ovarian cancer
Zhang Y *et al*., 2010, China	942 cases 1721 healthy hospital controls Age NA	2004–2008	Medical record	≥140/≥90 mm/Hg	Hypertension, all	Yes vs. no	6.34 (4.53–8.88)	Age
					Hypertension, type 1 endometrial cancer	Yes vs. no	6.39 (4.50–9.06)	
					Hypertension, type 2 endometrial cancer	Yes vs. no	6.63 (4.01–10.94)	
Friedenreich CM *et al*., 2011, Canada	515 cases 962 population controls Age 30–79 years	2002–2006	Self-reported (interview)	Not available	Ever diagnosed and treated for hypertension	Yes vs. no	1.75 (1.32–2.32)	Age, age^2^, age at menarche, number of pregnancies ≥20 weeks gestation, type of HRT, waist circumference, triglycerides, HDL-cholesterol, fasting blood glucose
Rosato V *et al*., 2011, Italy	454 cases 798 hospital controls Age 18–79/19–79 years	1992–2006	Self-reported (interview)	Not available	Hypertension	Yes vs. no	1.77 (1.34–2.34)	Age, study center, year of interview, education, age at menarche, parity, menopausal status, OC use, HRT use
Trabert B *et al*., 2015, USA	19323 cases 100751 population controls Age ≥65 years	1993–2007	Medical records	Not available	Hypertension	Yes vs. no	1.21 (1.15–1.26)	Age, diagnosis date, race/ethnicity, registry area, tobacco use, overweight/obesity, impaired fasting glucose, high triglycerides
Shao Y *et al*., 2015, China	128 cases 294 hospital controls Age 22–43 years	2010–2013	Self-reported (interview)	Not available	Hypertension	Yes vs. no	2.62 (0.90–4.40)	Age, time of day of blood collection, CRP, IL-6, TNF-α, insulin, C-peptide, SHBG, birth weight > 4 kg, BMI, WHR, diabetes, age at menarche, FH - cancer

ACE-inhibitor = angiotensin-converting enzyme inhibitor, BMI = body mass index, CRP = C-reactive protein, FH – EC = family history of endometrial cancer, HDL-cholesterol = high-density lipoprotein cholesterol, HRT use = hormone replacement therapy use, IL-6 = interleukin-6, NA = not available, OC use = oral contraceptive use, TNF-α = tumor necrosis factor α, WHR = waist-to-hip ratio.

**Table 3:** Subgroup analyses of hypertension and endometrial cancer.

		Hypertension and endometrial cancer				
		*n*	Relative risk (95% CI)	*I*^2^ (%)	*P*_h_^1^	*P*_h_^2^
All studies		25	1.61 (1.41–1.85)	86.3	<0.0001	
Cohort studies		6	1.32 (1.12–1.56)	47.4	0.09	0.21
Case-control studies		19	1.73 (1.45–2.06)	89.1	<0.0001	
Duration of follow-up (cohort studies)						
<10 years		1	1.50 (0.85–2.64)			0.72
≥10 years		5	1.31 (1.09–1.57)	57.0	0.05	
Geographic location						
Europe		7	1.68 (1.29–2.20)	81.5	<0.0001	0.33
America		14	1.38 (1.24–1.55)	69.6	<0.0001	
Asia		4	2.61 (1.08–6.33)	89.9	<0.0001	
Number of cases						
<250		11	1.77 (1.52–2.06)	11.2	0.34	0.63
250–<500		7	1.41 (1.10–1.81)	82.4	<0.0001	
≥500		7	1.64 (1.27–2.12)	94.5	<0.0001	
Exclusion of prevalent hysterectomies and/or censoring of incident hysterectomies						
Yes		16	1.51 (1.28–1.78)	88.5	<0.0001	0.32
No		9	1.81 (1.49–2.20)	56.5	0.02	
Study quality						
0–3 points		0				0.05
4–6		6	2.17 (1.38–3.40)	91.1	<0.0001	
7–9		19	1.43 (1.27–1.60)	73.8	<0.0001	
Adjustment for confounding factors^3^						
Age	Yes	25	1.61 (1.41–1.85)	86.3	<0.0001	NC
	No	0				
Smoking	Yes	9	1.26 (1.13–1.40)	58.2	0.01	0.02
	No	16	1.93 (1.52–2.45)	86.5	<0.0001	
Diabetes mellitus	Yes	10	1.56 (1.21–2.02)	77.2	<0.0001	0.78
	No	15	1.65 (1.39–1.97)	89.7	<0.0001	
BMI	Yes	15	1.27 (1.16–1.40)	54.3	0.006	0.003
	No	10	2.15 (1.62–2.86)	85.6	<0.0001	
Physical activity	Yes	3	1.27 (0.88–1.81)	62.8	0.07	0.35
	No	22	1.66 (1.44–1.93)	87.5	<0.0001	
Oral contraceptive use	Yes	7	1.23 (1.06–1.44)	61.4	0.02	0.02
	No	18	1.86 (1.53–2.25)	89.3	< 0.0001	
Hormone replacement therapy	Yes	9	1.34 (1.11–1.63)	81.4	< 0.0001	0.08
	No	16	1.84 (1.50–2.27)	88.6	< 0.0001	
Age at menarche	Yes	8	1.48 (1.15–1.91)	83.4	< 0.0001	0.49
	No	17	1.69 (1.42–2.02)	87.9	< 0.0001	
Parity	Yes	11	1.33 (1.16–1.54)	59.7	0.006	0.03
	No	14	1.93 (1.55–2.41)	91.3	< 0.0001	
Age at menopause	Yes	3	1.14 (1.01–1.30)	0	0.38	0.07
	No	22	1.72 (1.47–2.02)	87.5	< 0.0001	
Menopausal status	Yes	3	1.40 (1.03–1.89)	76.8	0.01	0.49
	No	22	1.66 (1.42–1.93)	87.3	< 0.0001	

*n* denotes the number of studies, ^1^P for heterogeneity within each subgroup, ^2^P for heterogeneity between subgroups with meta-regression analysis. NC, not calculable because no studies were present in one of the subgroups.
