# Corrigendum: Hypertension and the risk of endometrial cancer: a systematic review and meta-analysis of case-control and cohort studies

**DOI:** 10.1038/srep46961

**Published:** 2018-04-06

**Authors:** Dagfinn Aune, Abhijit Sen, Lars J. Vatten

Scientific Reports 7: Article number: 44808; 10.1038/srep44808 published online: 04 07 2017; updated: 04 06 2018.

In this Article, Abhijit Sen and Lars J. Vatten are incorrectly listed with ‘Department of Epidemiology and Biostatistics, Imperial College, London, UK’. The correct affiliation for these authors is listed below:

Department of Public Health and General Practice, Faculty of Medicine, Norwegian University of Science and Technology, Trondheim, Norway.

