# Supplementary material for: Hypertension and the risk of endometrial cancer: a systematic review and meta-analysis of case-control and cohort studies
Source: Sci Rep. 2017 Apr 7;7:44808. doi: 10.1038/srep44808 (PMC5384280; doi:10.1038/srep44808)
Supplement: Supplementary Information [file srep44808-s1.doc]

Supplementary information for:

Aune D, Sen A, Vatten LJ. Hypertension and the risk of endometrial cancer: a systematic review and meta-analysis of case-control and cohort studies. Scientific Reports 2017

Supplementary Table 1. Hypertension and endometrial cancer

| Exclusion reason | Reference number |
| --- | --- |
| Abstract | (1;2) |
| Duplicate | (3-5) |
| Elevated blood pressure as exposure (not hypertension) | (6) |
| No risk estimates | (7-9) |
| Not relevant exposure | (10-14) |
| Patients with pelvic inflammatory disease | (15) |
| Pooled analysis | (16;17) |
| Survival from endometrial cancer as outcome | (18) |

Reference List

1. Friedenreich CM, Lau DCW, Csizmadi I et al. Metabolic syndrome increases risk for endometrial cancer. American Journal of Epidemiology Conference: 3rd North American Congress of Epidemiology Montreal, QC Canada Conference Start: 2011;01.

2. Friedenreich CM, Langley AR, Lau W et al. Case-control study of inflammatory markers and endometrial cancer. American Journal of Epidemiology Conference: 45th Annual Meeting of the Society for Epidemiologic Research, SER 2012;15.

3. Folsom AR, Kaye SA, Potter JD, Prineas RJ. Association of incident carcinoma of the endometrium with body weight and fat distribution in older women: early findings of the Iowa Women's Health Study. Cancer Res 1989;49:6828-31.

4. Friedenreich CM, Langley AR, Speidel TP et al. Case-control study of markers of insulin resistance and endometrial cancer risk. Endocr Relat Cancer 2012;19:785-92.

5. La Vecchia C, Decarli A, Fasoli M, Gentile A. Nutrition and diet in the etiology of endometrial cancer. Cancer 1986;57:1248-53.

6. Cust AE, Kaaks R, Friedenreich C et al. Metabolic syndrome, plasma lipid, lipoprotein and glucose levels, and endometrial cancer risk in the European Prospective Investigation into Cancer and Nutrition (EPIC). Endocr Relat Cancer 2007;14:755-67.

7. Parazzini F, Negri E, La VC et al. Role of reproductive factors on the risk of endometrial cancer. International Journal of Cancer 76 (6) (pp 784-786), 1998;10.

8. Yu HC, Lin CY, Chang WC, Shen BJ, Chang WP, Chuang CM. Increased association between endometriosis and endometrial cancer: a nationwide population-based retrospective cohort study. Int J Gynecol Cancer 2015;25:447-52.

9. Filomeno M, Bosetti C, Bidoli E et al. Mediterranean diet and risk of endometrial cancer: a pooled analysis of three Italian case-control studies. Br J Cancer 2015;112:1816-21.

10. Weiderpass E, Sandin S, Lof M et al. Endometrial cancer in relation to coffee, tea, and caffeine consumption: a prospective cohort study among middle-aged women in Sweden. Nutr Cancer 2014;66:1132-43.

11. Hippisley-Cox J, Coupland C. Development and validation of risk prediction algorithms to estimate future risk of common cancers in men and women: prospective cohort study. BMJ Open 2015;5:e007825.

12. Gao J, Yang G, Wen W et al. Impact of known risk factors on endometrial cancer burden in Chinese women. Eur J Cancer Prev 2015.

13. Burghaus S, Haberle L, Schrauder MG et al. Endometriosis as a risk factor for ovarian or endometrial cancer - results of a hospital-based case-control study. BMC Cancer 2015;15:751.

14. Husing A, Dossus L, Ferrari P et al. An epidemiological model for prediction of endometrial cancer risk in Europe. Eur J Epidemiol 2016;31:51-60.

15. Yang TK, Chung CJ, Chung SD, Muo CH, Chang CH, Huang CY. Risk of Endometrial Cancer in Women With Pelvic Inflammatory Disease: A Nationwide Population-Based Retrospective Cohort Study. Medicine (Baltimore) 2015;94:e1278.

16. Cote ML, Alhajj T, Ruterbusch JJ et al. Risk factors for endometrial cancer in black and white women: a pooled analysis from the Epidemiology of Endometrial Cancer Consortium (E2C2). Cancer Causes Control 2015;26:287-96.

17. Stocks T, van HM, Manjer J et al. Blood pressure and risk of cancer incidence and mortality in the Metabolic Syndrome and Cancer Project. Hypertension 2012;59:802-10.

18. Nevadunsky NS, Van AA, Strickler HD et al. Metformin use and endometrial cancer survival. Gynecol Oncol 2014;132:236-40.

Supplementary Table 2. Influence analysis for hypertension and endometrial cancer

------------------------------------------------------------------------------

Study omitted | Estimate [95% Conf. Interval]

-------------------+----------------------------------------------------------

Elwood, 1977 | 1.6101284 1.4017038 1.8495444

Austin, 1991 | 1.5862452 1.3836886 1.8184539

Inoue, 1994 | 1.6120967 1.405624 1.8488981

Goodman, 1997 | 1.640528 1.4261634 1.8871135

Hachisuga, 1998 | 1.6189623 1.4083273 1.8611007

Soler, 1999 | 1.6160268 1.4015937 1.8632666

McCann, 2000 | 1.6049936 1.3963217 1.8448504

Salazar-Martinez, 2000| 1.5995344 1.3938072 1.835627

Weiderpass, 2000 | 1.6490747 1.428781 1.9033338

Strom, 2006 | 1.620527 1.4052563 1.868775

Weiss, 2006 | 1.6486589 1.4243011 1.9083579

Soliman, 2006 | 1.5911791 1.3880221 1.8240713

Fortuny, 2009 | 1.6467054 1.4338714 1.891131

Reis, 2009 | 1.562569 1.3692566 1.7831736

Zhang, 2010 | 1.4856873 1.3377228 1.6500179

Friedenreich, 2011| 1.6068733 1.397174 1.8480461

Rosato, 2011 | 1.6058804 1.3964452 1.8467263

Trabert, 2015 | 1.6520244 1.4075532 1.9389566

Shao, 2015 | 1.5968815 1.3928999 1.830735

Mack, 1976 | 1.6165415 1.407445 1.8567023

Tulinius, 1997 | 1.6029222 1.3957076 1.840901

Folsom, 2000 | 1.6198444 1.4039197 1.8689786

Furberg, 2003 | 1.6359873 1.4223026 1.8817757

Ollberding, 2011 | 1.6390138 1.4187902 1.8934203

Sponholtz, 2016 | 1.6501386 1.4338629 1.8990363

-------------------+----------------------------------------------------------

Combined | 1.612517 1.4077938 1.8470112

------------------------------------------------------------------------------
